# Markers of neutrophil mediated inflammation associate with disturbed continuous electroencephalogram after out of hospital cardiac arrest

**DOI:** 10.1111/aas.14145

**Published:** 2022-09-12

**Authors:** Pirkka T. Pekkarinen, Federico Carbone, Silvia Minetti, Davide Ramoni, Giuseppe Ristagno, Roberto Latini, Lauri Wihersaari, Kaj Blennow, Henrik Zetterberg, Jussi Toppila, Pekka Jakkula, Matti Reinikainen, Fabrizio Montecucco, Markus B. Skrifvars

**Affiliations:** ^1^ Division of Intensive Care, Department of Anaesthesiology, Intensive Care and Pain Medicine University of Helsinki and Helsinki University Hospital Helsinki Finland; ^2^ Department of Internal Medicine IRCCS Ospedale Policlinico San Martino Genoa–Italian Cardiovascular Network Genoa Italy; ^3^ First Clinic of Internal Medicine, Department of Internal Medicine University of Genoa Genoa Italy; ^4^ Department of Pathophysiology and Transplantation University of Milan Milan Italy; ^5^ Department of Anesthesiology, Intensive Care and Emergency Fondazione IRCCS Ca' Granda Ospedale Maggiore Policlinico Milan Italy; ^6^ Cardiovascular Medicine Mario Negri Institute for Pharmacological Research IRCCS Milan Italy; ^7^ Department of Anaesthesiology and Intensive Care Kuopio University Hospital and University of Eastern Finland Kuopio Finland; ^8^ Department of Psychiatry and Neurochemistry, Institute of Neuroscience and Physiology The Sahlgrenska Academy at the University of Gothenburg Mölndal Sweden; ^9^ Clinical Neurochemistry Laboratory Sahlgrenska University Hospital Mölndal Sweden; ^10^ Department of Neurodegenerative Disease UCL Institute of Neurology, Queen Square London UK; ^11^ UK Dementia Research Institute at UCL London UK; ^12^ Hong Kong Center for Neurodegenerative Diseases Hong Kong China; ^13^ Department of Clinical Neurophysiology, Medical Imaging Center, Helsinki University Central Hospital and Department of Clinical Neurosciences (Neurophysiology) University of Helsinki Helsinki Finland; ^14^ Department of Emergency Care and Services University of Helsinki and Helsinki University Hospital Helsinki Finland

**Keywords:** cardiac arrest, continuous electroencephalogram, hypoxia, inflammation, ischemia, neutrophilic granulocyte, postcardiac arrest syndrome, prognostication, reperfusion injury, seizures

## Abstract

**Background:**

Achieving an acceptable neurological outcome in cardiac arrest survivors remains challenging. Ischemia‐reperfusion injury induces inflammation, which may cause secondary neurological damage. We studied the association of ICU admission levels of inflammatory biomarkers with disturbed 48‐hour continuous electroencephalogram (cEEG), and the association of the daily levels of these markers up to 72 h with poor 6‐month neurological outcome.

**Methods:**

This is an observational, post hoc sub‐study of the COMACARE trial. We measured serum concentrations of procalcitonin (PCT), high‐sensitivity C‐reactive protein (hsCRP), osteopontin (OPN), myeloperoxidase (MPO), resistin, and proprotein convertase subtilisin/kexin type 9 (PCSK9) in 112 unconscious, mechanically ventilated ICU‐treated adult OHCA survivors with initial shockable rhythm. We used grading of 48‐hour cEEG monitoring as a measure for the severity of the early neurological disturbance. We defined 6‐month cerebral performance category (CPC) 1–2 as good and CPC 3–5 as poor long‐term neurological outcome. We compared the prognostic value of biomarkers for 6‐month neurological outcome to neurofilament light (NFL) measured at 48 h.

**Results:**

Higher OPN (*p* = .03), MPO (*p* < .01), and resistin (*p* = .01) concentrations at ICU admission were associated with poor grade 48‐hour cEEG. Higher levels of ICU admission OPN (OR 3.18; 95% CI 1.25–8.11 per ln[ng/ml]) and MPO (OR 2.34; 95% CI 1.30–4.21) were independently associated with poor 48‐hour cEEG in a multivariable logistic regression model. Poor 6‐month neurological outcome was more common in the poor cEEG group (63% vs. 19% *p* < .001, respectively). We found a significant fixed effect of poor 6‐month neurological outcome on concentrations of PCT (F = 7.7, *p* < .01), hsCRP (F = 4.0, *p* < .05), and OPN (F = 5.6, *p* < .05) measured daily from ICU admission to 72 h. However, the biomarkers did not have independent predictive value for poor 6‐month outcome in a multivariable logistic regression model with 48‐hour NFL.

**Conclusion:**

Elevated ICU admission levels of OPN and MPO predicted disturbances in cEEG during the subsequent 48 h after cardiac arrest. Thus, they may provide early information about the risk of secondary neurological damage. However, the studied inflammatory markers had little value for long‐term prognostication compared to 48‐hour NFL.

**Editorial comment**

These findings show that new biomarkers and continuous EEG are disturbed following successful resuscitation after cardiac arrest at the ICU. However, these parameters alone do not add to the prediction of long‐term outcomes. Thus, established markers and especially time and neurological assessments remain hallmarks in the prediction of outcome in postcardiac arrest patients.

## 1 INTRODUCTION

International Scientific Societies have recently highlighted the epidemiological relevance of sudden cardiac arrest (SCA). Although many variations due to different healthcare structures, processes of care and quality of medical treatment, SCA is the third leading cause of death in Europe, with an incidence ranging from 67 to 170 per 100,000 inhabitants.^1^ Surviving SCA with poor neurological outcome entails significant morbidity, and even survivors with good neurological outcome often suffer from neuro‐cognitive, fatigue and emotional problems.^1^ Prognostication after SCA requires a multimodal approach and, with the contemporary means, can be performed with reasonable accuracy at 72 h after SCA.^2^ Therefore, a biomarker that would have reliable prognostic value already at ICU admission, and optimally, potential to guide treatment is yet to be found. Since secondary neurological damage during postarrest care is largely associated to inflammation,^3^ inflammatory biomarkers have potential to fill this gap.

Here, we focused on molecules that have been recently associated with neutrophil infiltration to tissues (i.e., osteopontin [OPN])^4^ and neutrophil activation (i.e., myeloperoxidase [MPO] and resistin).^5^, ^6^ Neutrophils are early activated during ischemia/reperfusion, but their role was recently updated as critical in both injury and repair. Rather, a timely resolution of neutrophil inflammation is critical to orchestrate healing and tissue remodeling by triggering monocyte recruitment and their polarization into macrophages.^5^, ^7^ Proprotein convertase subtilisin/kexin type 9 (PCSK9), a marker of local vascular inflammation in coronary artery disease,^8^ and classical inflammatory markers procalcitonin (PCT) and high‐sensitivity C‐reactive protein (hsCRP) were also studied. Primary outcome of this study was then to investigate the association of the studied inflammatory biomarkers with early neurological disturbance defined as poor grade continuous electroencephalogram (cEEG) recording during the first 48 h after SCA. Based on preclinical data linking neuroinflammation with seizures after experimental cardiac arrest^7^ and the proposed role of inflammation in the pathophysiology of brain injury after SCA,^3^, ^9^ we hypothesized that ongoing inflammation in the CNS leading to secondary neurological damage would be reflected as disturbances in cEEG. Furthermore, we tested if these markers had predictive value for long‐term neurological outcome after SCA. Since a previous publication from the same patient cohort reported neurofilament light (NFL) measured at 48 h to be highly accurate in predicting 6‐month neurological outcome,^10^ we asked if the inflammatory markers would bring additional predictive value compared to it. NFL is a cytoskeletal neuron‐specific protein released to circulation after neuron death.^10^

## 2 METHODS

### 2.1 Study setting

The current study was a post hoc sub‐study of the COMACARE study (NCT02698917).^11^ The study included adult (age 18–80 years) patients with ventricular fibrillation (VF) or ‐tachycardia (VT) as initial rhythm causing out‐of‐hospital cardiac arrest (OHCA) admitted to the participating ICUs. Only patients with (suspected) cardiac cause of the arrest were included. The inclusion criteria required the patients to be unconscious, mechanically ventilated, and have return of spontaneous circulation (ROSC) between 10–45 min from the collapse. All study patients were subjected to targeted temperature management of 33 or 36°C for 24 h. The study patients were randomly assigned in a 2^3^ factorial design to lower or higher treatment targets of arterial blood partial pressure of oxygen (PaO_2_; low 10–15 kPa and high 20–25 kPa), arterial blood partial pressure of carbon dioxide (PaCO_2_; low 4.5–4.7 kPa and high 5.8–6.0 kPa) and mean arterial blood pressure (MAP; low 65–75 mmHg and high 80–100 mmHg). These treatment targets were maintained for the first 36 h of ICU stay. The primary outcome of the COMACARE study was serum neuron‐specific enolase (NSE) concentration 48 h after cardiac arrest and cEEG grading according to Crepeau et al.^12^ for the first 48 h, and Cerebral Performance Category (CPC) at 6 months after cardiac arrest were secondary outcome measures. The Research Ethics Committee of the Northern Savo Hospital District approved the study protocol (295/13.02.00/2015 §53). The study was conducted in accordance with the Declaration of Helsinki. We obtained deferred written informed consent from the next of kin and additionally from all those patients who recovered to sufficient neurological function enabling independent decision‐making.

### 2.2 Laboratory analysis

We used samples collected from 112 patients enrolled in the COMACARE‐trial in the six participating Finnish ICUs. The samples were drawn on ICU admission (median [IQR] delay from collapse to admission sample was 200 [160–230] minutes) and 24, 48, and 72 h after OHCA. We measured serum levels of PCT, hsCRP, OPN, MPO, resistin and PCSK9 by colorimetric enzyme‐linked immunosorbent assay (ELISA) following the manufacturer's instructions (PCT: Thermo Scientific, Frederick, MD, others: R&D Systems, Minneapolis, MN). The lowest detection thresholds were: 30 pg/ml for PCT, 15.625 pg/ml for CRP, 62.5 pg/ml for MPO and OPN, 31.25 pg/ml for resistin and 125 pg/ml for PCSK9. No samples fell below the detection thresholds. Intra‐ and inter‐assay coefficients of variation were below 8% for all markers. Previously published data on NSE^13^ and NFL^10^ levels from the same patient cohort were used for comparison in multivariable models.

### 2.3 Neurological outcome

We used grading of cEEG monitoring for the first 48 h after ICU admission to the ICU interpreted by an experienced senior neurophysiologist^11^ as a surrogate measure for the severity of the early neurological disturbance. The grading was performed as suggested by Crepeau et al.^12^ for cardiac arrest patients subjected to targeted temperature management. We defined grade 1 (excess beta, theta slowing and/or anesthetic pattern) as good early cEEG result, and grade 2 (diffuse local slowing, spindle coma, interictal epileptiform discharges etc.) and grade 3 (burst suppression pattern, focal or generalized seizures, status epilepticus etc.) as poor early cEEG result. We used CPC at 6 months after the cardiac arrest as long‐term neurological outcome measure. An experienced neurologist assessed patient's CPC class based on patient records and a telephone interview. We defined 6‐month CPC 1–2 as good and CPC 3–5 as poor neurological outcome.^14^

### 2.4 Statistics

To assess group differences, we applied the Student's *t* test (continuous variables) or Pearson Chi‐Square test (categorial variables). Due to right‐skewed distributions, we used log‐transformed values (ln[concentration]) of all the studied inflammatory biomarkers, NSE and NFL for the statistical analyses. We studied the association of biomarker concentrations with outcome using receiver operated characteristic curves (ROC) with area under the curve (AUC) analysis.

We built multivariable logistic regression models to evaluate the independent predictive value of biomarker concentrations for poor grade cEEG recoding of the first 48 h after OHCA. The following baseline confounding variables were considered for inclusion to the models: age, sex, bystander‐initiated resuscitation, delay to arrival of first unit, delay to ROSC and norepinephrine dose at ICU admission. The variables that were associated with the outcome variable at a *p* < .2 in univariate analysis were added to the multivariable model. ICU admission concentration of lactate, NSE, and NFL were then added to the model together with each studied biomarker.

To analyze the association between poor 6‐month neurological outcome and temporal change in biomarker concentrations, and between PaCO_2_, PaO_2_ and MAP intervention groups and temporal change in biomarker concentrations, we employed linear models with alternative covariance structures and restricted maximum likelihood (REML) estimation. We used the MIXED procedure of the SPSS program. The method allows analysis of repeated measures and tolerates occasional missing values.^15^ We applied unstructured covariance matrix in the models, because it provided the lowest Akaike's information criterion (AIC) in all models used. We assessed significant effects and interactions with least‐significant‐difference tests. The residuals from the models were visually determined to follow normal distribution, confirming that the model assumptions were reasonably met.

We built multivariable regression models to assess the independent predictive value of biomarker concentrations for predicting poor 6‐month neurological outcome. Based on our previous results indicating NFL at 48 h having high discriminatory potential for poor 6‐month neurological outcome,^10^ we focused on finding variables that would have additional predictive potential when used together with NFL. The studied biomarkers with statistical significance in ROCAUC analysis with the lowest P value for predicting 6‐month neurological outcome were added separately to the model with 48‐hour NFL.

We performed statistical analyses with the SPSS software (version 27.0, IBM, Armonk, NY, USA). We used GraphPad Prism software, version 9, https://www.graphpad.com to draw the figures.

## 3 RESULTS

In total, 112 patients were enrolled during the recruitation period March 22, 2016 to November 3, 2017. Of these, 111, 111, 109, and 106 patients had sera available for measurement of biomarkers at ICU admission, 24, 48, and 72 h, respectively. One patient had a missing ICU admission sample and another one a missing 72‐hour sample due to sampling error. Other missing samples were caused by early deaths or treatment withdrawals. At 30 days after OHCA, 36 patients were dead, and 76 patients were alive. Of the 30‐day survivors, one patient died before 6‐month follow‐up and two patients had severe cerebral disability (CPC 3) 6 months after OHCA. The remaining 73 patients had good 6‐month neurological outcome (CPC 1 and 2, 53 and 20 patients, respectively). No patient was lost to follow‐up. The median (IQR) ICU admission concentrations of biomarkers were: PCT 0.12 (0.10–0.20) ng/ml, hsCRP 0.83 (0.38–2.1) μg/ml, OPN 53 (41–79) ng/ml, MPO 97 (50–190) ng/ml, Resistin 40 (28–59) ng/ml and PCSK9 160 (100–210) ng/ml. Characteristics of the study population are presented in Table 1.

**TABLE 1:** Characteristics

	All *N* = 112	Good cEEG *N* = 72	Poor cEEG *N* = 38	*p* value
Age (years)	62 (53–68)	60 (50–67)	66 (57–74)	.01^a^
Sex (male)	82%	86%	76%	.29
Bystander initiated resuscitation (yes)	83%	81%	90%	.29
Time to first unit (min)	7 (6–9)	7 (5–9)	8 (7–10)	.02^a^
Time to ROSC^c^ (min)	21 (16–26)	18 (15–24)	25 (21–30)	<.001^b^
APACHE II (point)	28 (24–31)	27 (24–30)	30 (26–35)	<.001^b^
Norepinephrine at ICU arrival (μg/kg/min)	0.02 (0.00–0.12)	0.04 (0.00–0.13)	0.01 (0.00–0.11)	.73
Admission lactate (mmol/L)	2.3 (1.3–3.4)	2.1 (1.3–3.3)	2.6 (1.5–3.7)	.14
Highest lactate of first 48 h (mmol/L)	2.5 (1.7–3.9)	2.4 (1.6–3.4)	2.9 (2.1–4.7)	.04^a^
NFL at 48 h (ng/ml)	30 (15–610)	24 (14–49)	1800 (32–4800)	<.001^b^
NSE at 48 h (ng/ml)	22 (14–34)	19 (13–27)	36 (22–126)	<.001^b^
Poor 6‐month neurological outcome	35%	19%	63%	<.001^b^
Poor 48‐hour cEEG	35%	N/A	N/A	

*Note*: For continuous variables, median (IQR) and P‐value from Mann Whitney U test for the difference between cEEG groups is presented; for categorical variables, percentage of the whole and *p* value from two‐tailed Fisher's Exact test is presented. Continuous 48‐hour EEG (cEEG) recording was graded as suggested by Crepeau et al.; we defined grade 1 as good and grade 2–3 as poor cEEG result. Good 6‐month neurological outcome was defined as Cerebral Performance Categories (CPC) 1–2; poor outcome as CPC 3–5; Two patients with poor cEEG result had missing 48‐hour NSE and NFL samples due to early treatment withdrawal. Two patients had missing cEGG.

^a^ Statistically significant at the *p* < .05 level.

^b^ Statistically significant at the *p* < .01 level.

^c^ Time to ROSC 10–45 min was an inclusion criterion for the COMACARE study.

### 3.1 Association of biomarker ICU admission values with poor grade continuous EEG of the first 48 h

**FIGURE 1:**
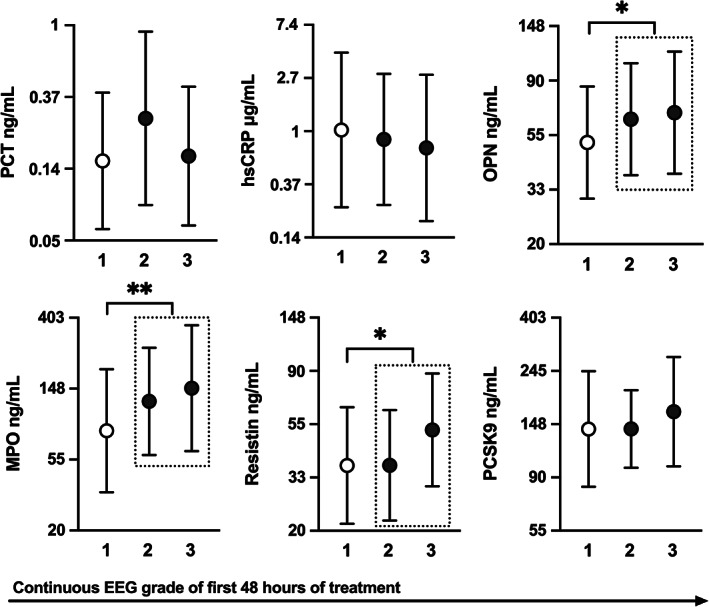
ICU admission biomarker concentrations in relation to poor grade continuous EEG (cEEG) recording of the first 48 h after OHCA. cEEG recording was graded as suggested by Crepeau et al.; we defined grade 1 as good cEEG result (open circles, *N* = 71), and grade 2 (*N* = 7) and grade 3 (*N* = 31) as poor cEEG result (closed circles). Mean values (circles) with SD (error bars) calculated from the ln transformed data are presented. Note the logarithmic scale. *Statistically significant at the *p* < .05 level. **Statistically significant at the *p* < .01 level. EEG, electroencephalogram; hsCRP, high‐sensitivity C‐reactive protein; MPO, myeloperoxidase; OPN, Osteopontin; PCT, Procalcitonin; PCSK9, Proprotein convertase subtilisin/kexin type 9

Thirty‐eight patients had poor grade cEEG (seven patients grade 2; 31 patients grade 3), and 71 patients had good grade (1) cEEG recording of the first 48 h after OHCA. Two patients had missing cEGG data. Poor 6‐month neurological outcome was more common in the poor cEEG group compared to the good cEEG group (63% vs. 19% *p* < .001, respectively). In the poor cEEG group, 18% of patients had good 6‐month neurological outcome with CPC 1 and 18% with CPC 2, whereas in the good cEEG group the distribution was 63% CPC 1 and 18% CPC 2. We tested if the ICU admission concentrations of the biomarkers were different between patients with poor or good cEEG. Higher OPN (*p* = .03), MPO (*p* < .01) and resistin (*p* = .01) concentrations at ICU admission were associated with poor grade cEEG. (Figure 1) In ROC analysis OPN (AUC 0.65; 95% CI 0.53–0.76), MPO (0.67; 0.56–0.77), and resistin (0.64; 0.54–0.75) had statistically significant predictive value for poor cEEG recording (Table 2).

**TABLE 2:** ROCAUC values (95% CI) of admission biomarker levels for predicting poor grade continuous EEG recording of the 48 h after OHCA

	AUC (95% CI)
PCT	0.55 (0.44–0.67)
hsCRP	0.43 (0.33–0.54)
OPN	0.65^a^ (0.53–0.76)
MPO	0.67^a^ (0.56–0.77)
Resistin	0.64^a^ (0.54–0.75)
PCSK9	0.58 (0.47–0.69)
NSE	0.48 (0.36–0.60)
NFL	0.55 (0.44–0.66)
Lactate	0.59 (0.48–0.70)

^a^ Statistically significant at the *p* < .05 level.

Finally, we assessed the independent predictive value of ICU admission concentrations of biomarkers in multivariable logistic regression models. The models were built adjusting for relevant clinical confounding factors that were associated with the outcome variable at a *p* < .2 in univariate analysis (age, delay to arrival of first unit and delay to ROSC) and ICU admission concentration of NSE, NFL, and lactate. Higher levels of ICU admission OPN, MPO and resistin were independently associated with poor cEEG in these models. (Table 3). Internal validation of the models with bootstrapping of 1000 random samples from the data supported the association of age (*p* < .05 in all models), delay to ROSC (*p* < .01 in all models), OPN (*p* = .02) and MPO (*p* < .01) with poor 48 h cEEG recording, whereas the association of resistin with the outcome variable was not significant after bootstrapping (*p* = .06).

**TABLE 3:** Logistic regression analysis for the association of admission biomarker levels with poor grade continuous EEG recording of the 48 h after OHCA

	OR	95% CI	*p*
Age (year)	1.09	1.02—1.16	.01^a^
First unit (min)	1.11	0.98—1.26	.11
ROSC (min)	1.14	1.06—1.23	<.01^b^
Lactate (mmol/L)	1.15	0.92—1.45	.22
NSE ln(ng/ml)	0.73	0.23—2.29	.59
NFL ln(ng/ml)	0.44	0.17—1.13	.09
PCT ln(ng/ml)	1.48	0.89—2.44	.13

	OR	95% CI	*p*
Age (year)	1.08	1.02—1.14	.01^a^
First unit (min)	1.06	0.94—1.21	.34
ROSC (min)	1.16	1.07—1.26	<.01^b^
Lactate (mmol/L)	1.14	0.91—1.42	.25
NSE ln(ng/ml)	0.63	0.20—2.01	.44
NFL ln(ng/ml)	0.53	0.20—1.37	.19
hsCRP ln(μg/ml)	0.78	0.55—1.11	.17

	OR	95% CI	*p*
Age (year)	1.09	1.02—1.16	.01^a^
First unit (min)	1.11	0.97—1.26	.13
ROSC (min)	1.15	1.06—1.24	<.01^b^
Lactate (mmol/L)	1.21	0.95—1.55	.13
NSE ln(ng/ml)	0.65	0.20—2.12	.48
NFL ln(ng/ml)	0.41	0.14—1.15	.09
OPN ln(ng/ml)	3.18	1.25—8.11	.02^a^

	OR	95% CI	*p*
Age (year)	1.09	1.02—1.16	.01^a^
First unit (min)	1.13	0.99—1.29	.07
ROSC (min)	1.13	1.05—1.22	<.01^b^
Lactate (mmol/L)	1.15	0.92—1.44	.22
NSE ln(ng/ml)	0.54	0.14—2.14	.38
NFL ln(ng/ml)	0.51	0.19—1.38	.18
MPO ln(ng/ml)	2.34	1.30—4.21	<.01^b^

	OR	95% CI	*p*
Age (year)	1.07	1.01—1.14	.02^a^
First unit (min)	1.11	0.98—1.26	.09
ROSC (min)	1.13	1.05—1.22	<.01^b^
Lactate (mmol/L)	1.16	0.93—1.46	.19
NSE ln(ng/ml)	0.62	0.19—2.04	.43
NFL ln(ng/ml)	0.49	0.18—1.33	.16
Resistin ln(ng/ml)	2.53	1.00—6.38	.05^a^

	OR	95% CI	*p*
Age (year)	1.08	1.02—1.14	.01^a^
First unit (min)	1.08	0.96—1.22	.21
ROSC (min)	1.15	1.06—1.24	<.01^b^
Lactate (mmol/L)	1.14	0.92—1.42	.24
NSE ln(ng/ml)	0.73	0.23—2.28	.59
NFL ln(ng/ml)	0.51	0.20—1.28	.15
PCSK9 ln(ng/ml)	1.46	0.59—3.61	.41

*Note*: Variables considered for inclusion in the models were: age, sex, bystander‐initiated resuscitation, delay to arrival of first unit, delay to ROSC and norepinephrine dose at ICU admission. Variables with a *p* < .2 in univariate analysis were added to the model. Admission lactate concentration, admission ln(concentration) of neuron specific enolase (NSE), admission ln(concentration) of neurofilament light (NFL) and each studied biomarker at a time were then entered to the model.

^a^ Statistically significant at the *p* < .05 level.

^b^ Statistically significant at the *p* < .01 level.

### 3.2 Association of 6‐month neurological outcome with biomarker levels

**FIGURE 2:**
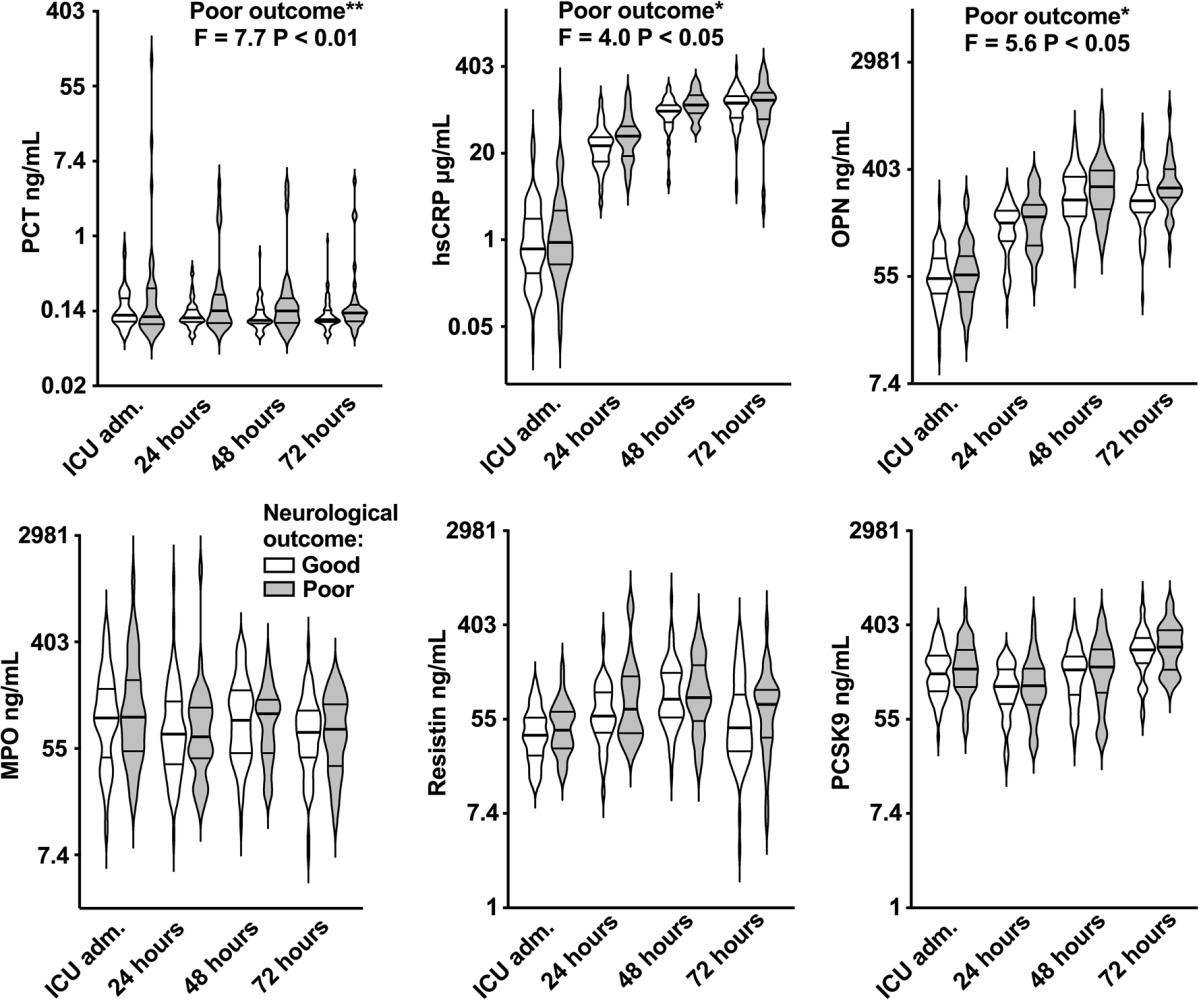
Biomarker concentrations in 6‐month neurological outcome groups. Violin plots with median (thick line) and IQR (thin lines) are presented. Note the logarithmic scale. ICU adm., ICU admission; Good neurological outcome, 6‐month Cerebral Performance Categories (CPC) 1–2; Poor outcome, 6‐month CPC 3–5. *Statistically significant at the *p* < .05 level. **Statistically significant at the *p* < .01 level. hsCRP, high‐sensitivity C‐reactive protein; MPO, myeloperoxidase; OPN, Osteopontin; PCT, Procalcitonin; PCSK9, Proprotein convertase subtilisin/kexin type 9

In the linear model analysis with REML estimation, the concentration of all studied biomarkers (PCT, hsCRP, OPN, MPO, resistin, PCSK9) changed as a function of time between the four measured timepoints from ICU admission to 72 h (statistically significant fixed effect of timepoint on biomarker concentration, *p* < .001 for all). We found a significant fixed effect of poor 6‐month neurological outcome on concentrations of PCT (F = 7.7, *p* < .01), hsCRP (F = 4.0, *p* < .05), and OPN (F = 5.6, *p* < .05). (Figure 2 and Table S1) The interaction term for timepoint × poor neurological outcome was not significant in any of these models. Biomarker concentrations in outcome groups are presented in Figure 2. We did not find a significant fixed effect of any of the intervention groups (treatment targets of PaCO_2_, PaO_2_, and MAP) on the concentrations of the biomarkers (Figure S1).

The predictive value for poor 6‐month neurological outcome of each biomarker at each studied timepoint was assessed as ROCAUC (Table 4). Next, we built multivariable logistic regression models to study the possible independent predictive value of biomarkers for poor 6‐month neurological outcome. We added biomarker concentrations on selected timepoints based on statistical significance with the lowest P value in ROCAUC analysis (namely, PCT at 48 h, hsCRP at 48 h and OPN at 72 h) to a multivariable model with 48‐hour NFL. The biomarkers did not have independent predictive value in the multivariable model with 48‐hour NFL (Table S2).

**TABLE 4:** Biomarker ROCAUC values (95% CI) for predicting poor outcome at studied timepoints

	Admission	24 h	48 h	72 h
PCT	0.50 (0.38–0.62)	0.61 (0.49–0.73)	0.62^a^ (0.50–0.74)	0.62 (0.50–0.73)
hsCRP	0.53 (0.41–0.64)	0.63^a^ (0.52–0.75)	0.67^a^ (0.56–0.78)	0.55 (0.43–0.67)
OPN	0.55 (0.43–0.66)	0.58 (0.46–0.69)	0.59 (0.48–0.71)	0.68^a^ (0.57–0.79)
MPO	0.52 (0.41–0.64)	0.49 (0.37–0.60)	0.47 (0.36–0.58)	0.51 (0.39–0.64)
Resistin	0.57 (0.46–0.69)	0.58 (0.46–0.70)	0.52 (0.40–0.64)	0.59 (0.47–0.70)
PCSK9	0.55 (0.44–0.67)	0.52 (0.40–0.64)	0.53 (0.41–0.65)	0.56 (0.44–0.68)

^a^ Statistically significant at the *p* < .05 level.

## 4 DISCUSSION

In this study, we show that in OHCA patients with successful resuscitation from VF/VT, ICU admission OPN and MPO are independent predictors for poor grade cEEG recorded during the following 48 h of ICU treatment. At ICU admission, their association with poor cEEG in multivariable logistic regression model was stronger than that of classical inflammatory markers PCT or hsCRP and markers of neurological injury, NSE or NFL. Furthermore, higher levels of OPN, PCT and hsCRP measured daily from ICU admission to 72 h were associated with poor 6‐month neurological outcome. However, they were not independent predictors of 6‐month neurological outcome when modeled with NFL measured at 48 h after arrest. From the clinical viewpoint, the overlapping distributions of the studied markers in 6‐month neurological outcome groups (Figure 2) discourage their use for prognostication and clinical decision‐making including withdrawal of treatment after OHCA with initial shockable rhythm (VF/VT). Our results should be interpreted as supporting evidence for the role of inflammation in the pathophysiology of secondary neurological damage during early postresuscitation care.

The rationale behind studying inflammatory biomarkers is that inflammation may precede and cause neurological damage. An early rising inflammatory marker could be used for identification of patients who could benefit from an anti‐inflammatory treatment intervention to prevent secondary neurological damage. The central nervous system (CNS) is particularly vulnerable to hypoxia during cardiac arrest due to its high metabolic rate and limited energy reserves.^16^ Hypoxic injury to the CNS leads to local release of leukocyte chemoattractant molecules,^17^ making it susceptible to infiltration of neutrophils and secondary inflammatory damage.^18^, ^19^ A recent preclinical study in rats demonstrated that the expression of multiple inflammatory mediators is increased in the CNS after cardiac arrest and that pharmacological increasing of these mediators increased seizure activity and worsened neurological outcome, whereas pharmacological reduction of the mediators had an opposite effect.^20^

Multiple studies have shown that elevated levels of inflammatory biomarkers in circulation are associated with poor outcome after cardiac arrest.^3^, ^21^, ^22^ This association may depend on case‐mix, being stronger in a patient population including more moribund patients^23^ compared to a more selected population.^24^ Interleukin‐6 and PCT are the most studied inflammatory markers in this context, and they have discriminatory capacity for poor outcome when measured at ICU admission^25^ and at 24 h after cardiac arrest,^26^ respectively. Failure of other organ systems than CNS is also common after cardiac arrest, has impact on outcome,^27^ and may be exacerbated by inflammation. Oxidative metabolism is disturbed in tissues during cardiac arrest and we have previously shown in the COMACARE cohort that FGF‐21, a marker of mitochondrial and cellular stress is associated with outcome after cardiac arrest.^28^ However, biomarkers should not to be used alone for outcome prediction, and the only biomarker recommended to be included in the multimodal approach for prognostication by current guidelines on postresuscitation care is NSE, a marker of CNS tissue destruction having acceptable prognostic value 48 h after cardiac arrest.^2^

Among the biomarkers here investigated, indirect–and partially controversial–data are available in both clinical and experimental models of brain ischemia–reperfusion injury (IRI).^5^, ^29^, ^30^, ^31^ Neutrophil recruitment and oxidative burst–of which OPN and MPO may be considered as surrogate markers, respectively–indeed represent a double edge sword. Alongside a detrimental effect in the early phase of IRI, OPN would exert a neuroprotective role mainly relying on its roles in the balancing of anti−/pro‐inflammatory responses, blood–brain barrier integrity as well as proliferation of active and the migration of resting astrocytes.^32^ In line with that, OPN is under investigation as potential candidate for therapeutic application in acute brain injury.^31^ Similarly, the amplification of oxidative burst sustained by neutrophil recruitment and activation may ultimately sustain neuronal recovery by both genetic and epigenetic mechanisms.^5^ What is not yet known is the extent to and the timing with inflammatory response shift from detrimental to beneficial for brain milieu.

In the current study, ICU admission values of neutrophil activation markers were associated with short‐term neurological disturbance reflected as seizure activity in the cEEG. This suggests that the detrimental neutrophil activation occurs within hours after ROSC. Once the brain tissue damage has occurred, structural proteins of neurons such as NFL are increasingly seen in the circulation at 24 and 48 h after ROSC,^10^ and the inflammatory markers did not contribute additional prognostic information for 6‐month neurological outcome compared to NFL measured at 48 h after ROSC in the current study.

The growing awareness about the complex network of interaction of different biological pathways is changing the attitude about the use of biomarkers, which are increasing pooled. The benefits of this approach clearly emerged from the COMMUNICATE study^33^ and has been recently supported by an exhaustive systematic review.^34^ Advance in machine/deep learning approach–including the use of artificial neural network–are paving the way for further advance prediction of OHCA‐related neurological outcome. An exploratory study from the Target Temperature Management trial finally allowed to exceed the 90% in the AUROC during the first 72 h after the event.^35^ More is expected from future studies, in which such advance in data mining will allow to merge traditional data (e.g., clinical, biochemical and imaging) with other relevant information from omics, data internet use, wearable devices and others: the so called “electronic health record”.

### 4.1 Strengths and limitations of the study

The study is a multicenter study performed in a government‐funded healthcare system with minimal variations of treatment between centers. No patient was lost to follow‐up and missing samples were rare. Outcome assessors were blinded to biomarker results and analysis of biomarkers was performed blinded to clinical data and outcome. The two major limitations of the study are the relatively small sample size and the enrollment of highly selected good prognosis patients (VF‐OHCA only, ROSC delay between 10 and 45 min, median APACHE II 28, good outcome 65%). Reperfusion injury is presumably greater in patients with initial non‐shockable rhythm and ROSC >45 min. These limitations prevented us from analyzing the impact of the cause of cardiac arrest (such as respiratory vs. cardiac, primary arrhythmic vs acute myocardial infarction) on biomarker levels. Therefore, our results need to be validated in future unselected cohorts–including patients with poorer prognosis due to other clinical causes of OHCA–in which inflammation likely plays a more important role.

## 5 CONCLUSIONS

In OHCA patients with successful resuscitation from VF/VT, high concentrations of OPN and MPO at ICU admission were independent predictors for disturbances in cEEG during the subsequent 48 h. Thus, these inflammatory markers may provide early information about the risk of secondary neurological damage. Higher levels of PCT, hsCRP and OPN measured daily from ICU admission to 72 h were associated with poor 6‐month neurological outcome but did not contribute independent prognostic value when modeled with NFL measured at 48 h.

## AUTHOR CONTRIBUTIONS

All listed authors meet the requirements of authorship and have approved the final version of the manuscript. Pirkka T. Pekkarinen, Federico Carbone, Fabrizio Montecucco, and Markus B. Skrifvars designed the study and wrote the manuscript. Pirkka T. Pekkarinen carried out data analysis and interpretation and drew the figures with major contributions from Markus B. Skrifvars All other authors (Silvia Minetti, Davide Ramoni, Giuseppe Ristagno, Roberto Latini, Lauri Wihersaari, Kaj Blennow, Henrik Zetterberg, Jussi Toppila, Pekka Jakkula, and Matti Reinikainen) made moderate contributions to the study design and manuscript revision.

## FUNDING INFORMATION

This study was funded by Helsinki University Hospital funding (project M7100YLIT2) and grants from Finska Läkaresällskapet and Einar och Karin Stroems stiftelse to Pirkka T. Pekkarinen; The funders had no role in study design, data collection, statistical analysis planning, manuscript writing or selection of the publication forum.

## CONFLICT OF INTEREST

Giuseppe Ristagno has served at data monitoring committee for DSMB trial: TRansfusion Strategies in Acute Brain INjured Patients (TRAIN).

Kaj Blennow has received consulting fees from Abcam, Axon, BioArctic, Biogen, JOMDD/Shimadzu, Lilly, MagQu, Prothena, Roche Diagnostics and Siemens Healthineers; honoraria for lectures from: GEECD/Roche Diagnostics and IFCC/SNIBE; has served at data monitoring committees for: Julius Clinical and Novartis and he is a co‐founder of Brain Biomarker Solutions in Gothenburg AB (BBS), which is a part of the GU Ventures Incubator Program.

Henrik Zetterberg has served at scientific advisory boards and/or as a consultant for Abbvie, Alector, Annexon, Artery Therapeutics, AZTherapies, CogRx, Denali, Eisai, Nervgen, Novo Nordisk, Pinteon Therapeutics, Red Abbey Labs, Passage Bio, Roche, Samumed, Siemens Healthineers, Triplet Therapeutics, and Wave, has given lectures in symposia sponsored by Cellectricon, Fujirebio, Alzecure, Biogen, and Roche, and is a co‐founder of Brain Biomarker Solutions in Gothenburg AB (BBS), which is a part of the GU Ventures Incubator Program.

Jussi Toppila has received consulting/lecture fees from: UCB Pharma Finland and SGS Fimko Ltd.

## Supporting information


**Figure S1** A, Biomarker concentrations in PaCO2 target groups.

**Figure S2** B, Biomarker concentrations in PaCO2 target groups.

**Figure S3** C, Biomarker concentrations in MAP target groups.

**Table S1** Fixed effect of poor neurological outcome on ln(concentration) of biomarkers (*n* = 112).

**Table S2** Logistic regression analysis for the association of biomarker levels with poor 6‐month neurological outcome.
