# Supplementary figures and images for: Markers of neutrophil mediated inflammation associate with disturbed continuous electroencephalogram after out of hospital cardiac arrest
Source: Acta Anaesthesiol Scand. 2022 Sep 12;67(1):94–103. doi: 10.1111/aas.14145 (PMC10087484; doi:10.1111/aas.14145)

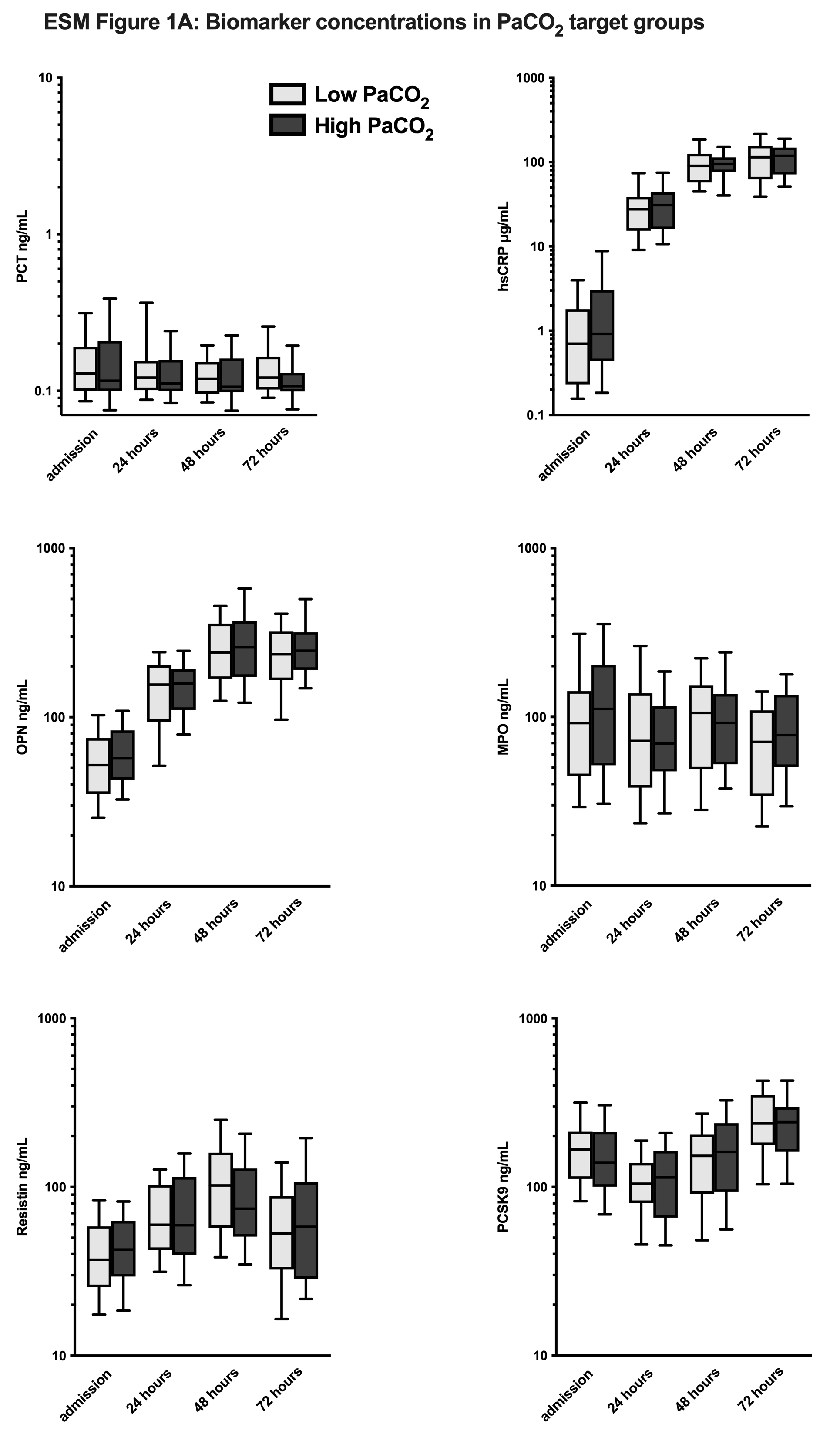

Supplement: Supplementary file 1 — Figure S1 A, Biomarker concentrations in PaCO2 target groups. [file AAS-67-94-s004.tiff]

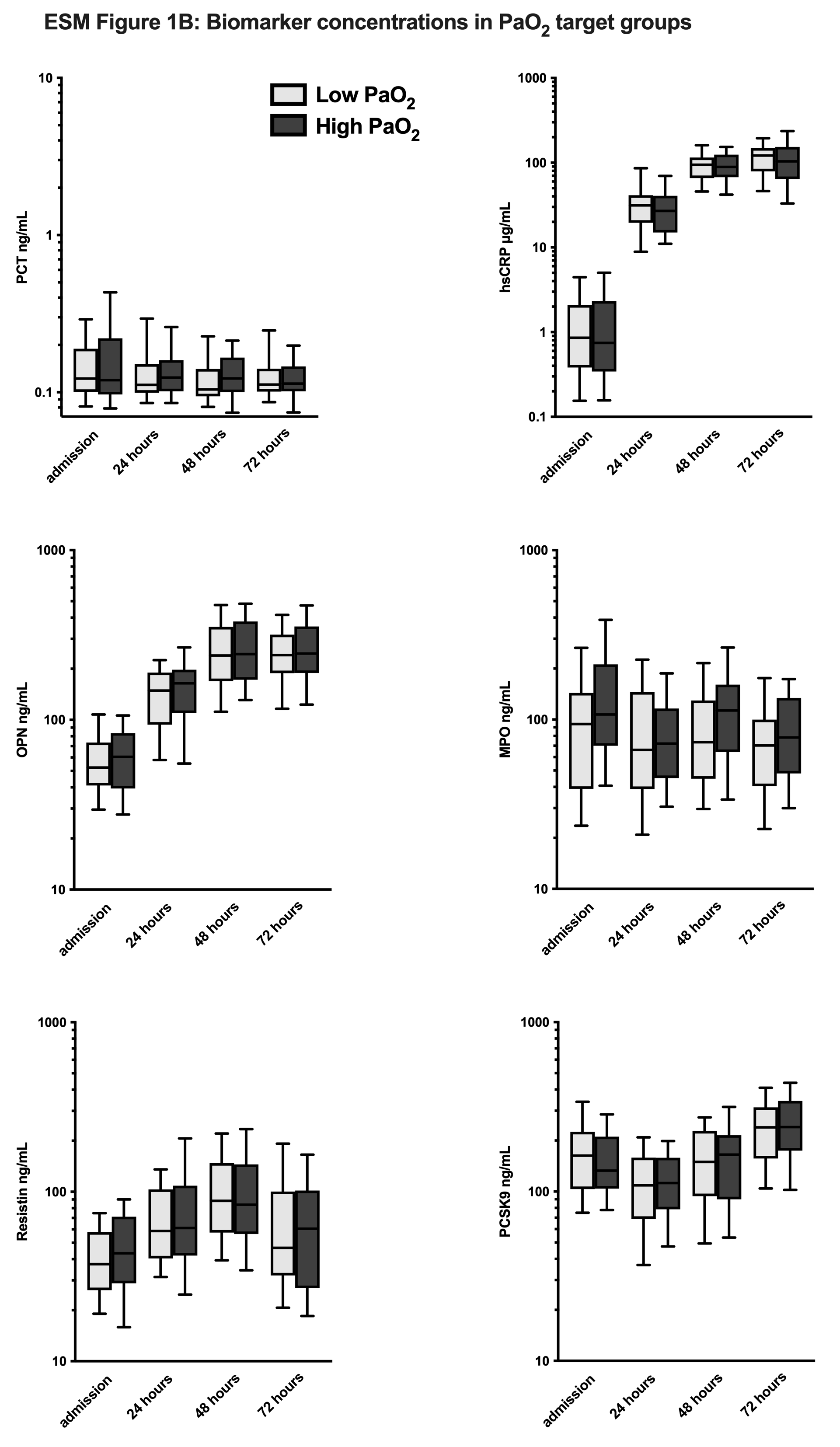

Supplement: Supplementary file 2 — Figure S2 B, Biomarker concentrations in PaCO2 target groups. [file AAS-67-94-s001.tiff]

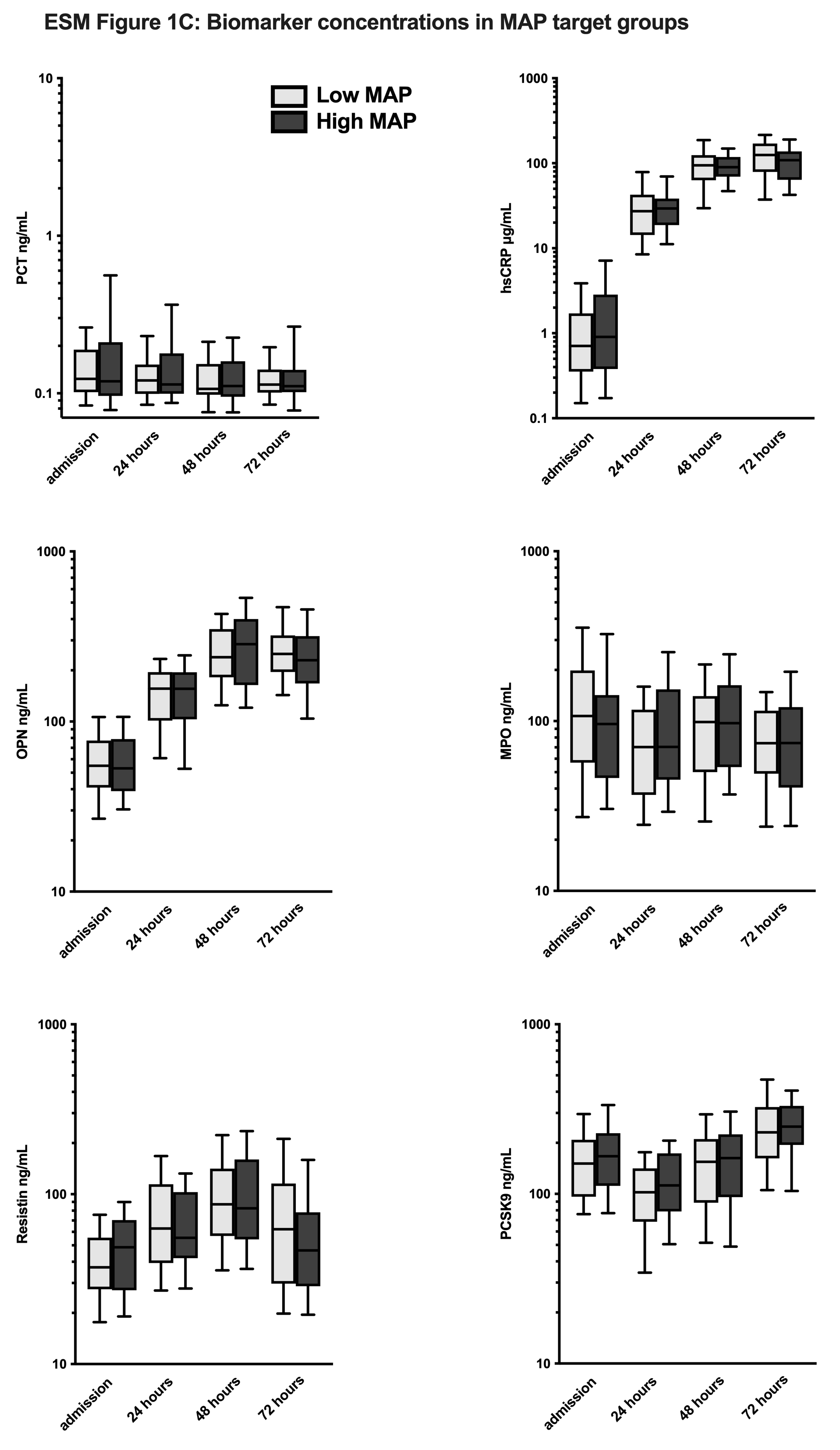

Supplement: Supplementary file 3 — Figure S3 C, Biomarker concentrations in MAP target groups. [file AAS-67-94-s002.tiff]
