# Supplementary material for: Markers of neutrophil mediated inflammation associate with disturbed continuous electroencephalogram after out of hospital cardiac arrest
Source: Acta Anaesthesiol Scand. 2022 Sep 12;67(1):94–103. doi: 10.1111/aas.14145 (PMC10087484; doi:10.1111/aas.14145)
Supplement: Supplementary file 4 — Table S1 Fixed effect of poor neurological outcome on ln(concentration) of biomarkers (n = 112). [file AAS-67-94-s005.docx]

ESM Table 1. Fixed effect of poor neurological outcome on ln(concentration) of biomarkers (n = 112)

|  | df | F | P-value |
| --- | --- | --- | --- |
| PCT | 1 | 7.7 | < 0.01** |
| hsCRP | 1 | 4.0 | 0.049* |
| OPN | 1 | 5.6 | 0.020* |
| MPO | 1 | 0.034 | 0.85 |
| Resistin | 1 | 2.9 | 0.091 |
| PCSK9 | 1 | 0.81 | 0.37 |
