# Supplementary material for: Markers of neutrophil mediated inflammation associate with disturbed continuous electroencephalogram after out of hospital cardiac arrest
Source: Acta Anaesthesiol Scand. 2022 Sep 12;67(1):94–103. doi: 10.1111/aas.14145 (PMC10087484; doi:10.1111/aas.14145)
Supplement: Supplementary file 5 — Table S2 Logistic regression analysis for the association of biomarker levels with poor 6‐month neurological outcome. [file AAS-67-94-s003.docx]

ESM Table 2. Logistic regression analysis for the association of biomarker levels with poor 6-month neurological outcome.

|  | OR |  | 95% CI |  | P |
| --- | --- | --- | --- | --- | --- |
| NFL 48 h ln(ng/mL) | 7.50 | 3.06 | - | 18.4 | <0.01** |
| PCT 48 h ln(ng/mL) | 0.74 | 0.16 | - | 3.42 | 0.74 |

|  | OR |  | 95% CI |  | P |
| --- | --- | --- | --- | --- | --- |
| NFL 48 h ln(ng/mL) | 8.22 | 2.98 | - | 22.7 | <0.01** |
| hsCRP 48 h ln(μg/mL) | 2.52 | 0.59 | - | 10.7 | 0.21 |

|  | OR |  | 95% CI |  | P |
| --- | --- | --- | --- | --- | --- |
| NFL 48 h ln(ng/mL) | 6.9 | 2.91 | - | 16.3 | <0.01** |
| OPN 72 h ln(ng/mL) | 1.47 | 0.18 | - | 12.0 | 0.72 |
